# Do nasogastric tubes worsen dysphagia in patients with acute stroke?

**DOI:** 10.1186/1471-2377-8-28

**Published:** 2008-07-23

**Authors:** Rainer Dziewas, Tobias Warnecke, Christina Hamacher, Stefan Oelenberg, Inga Teismann, Christopher Kraemer, Martin Ritter, Erich B Ringelstein, Wolf R Schaebitz

**Affiliations:** 1 Department of Neurology, University Hospital of Münster, Albert-Schweitzer-Straße 33, 48129 Münster, Germany

## Abstract

**Background:**

Early feeding via a nasogastric tube (NGT) is recommended as safe way of supplying nutrition in patients with acute dysphagic stroke. However, preliminary evidence suggests that NGTs themselves may interfere with swallowing physiology. In the present study we therefore investigated the impact of NGTs on swallowing function in acute stroke patients.

**Methods:**

In the first part of the study the incidence and consequences of pharyngeal misplacement of NGTs were examined in 100 stroke patients by fiberoptic endoscopic evaluation of swallowing (FEES). In the second part, the effect of correctly placed NGTs on swallowing function was evaluated by serially examining 25 individual patients with and without a NGT in place.

**Results:**

A correctly placed NGT did not cause a worsening of stroke-related dysphagia. Except for two cases, in which swallowing material got stuck to the NGT and penetrated into the laryngeal vestibule after the swallow, no changes of the amount of penetration and aspiration were noted with the NGT in place as compared to the no-tube condition. Pharyngeal misplacement of the NGT was identified in 5 of 100 patients. All these patients showed worsening of dysphagia caused by the malpositioned NGT with an increase of pre-, intra-, and postdeglutitive penetration.

**Conclusion:**

Based on these findings, there are no principle obstacles to start limited and supervised oral feeding in stroke patients with a NGT in place.

## Background

Dysphagia is an important complication of acute stroke. Abnormal lip closure, lingual incoordination, and delayed or absent triggering of the swallowing reflex may lead to a disturbance of both the oral and the pharyngeal phase of swallowing. In the acute stage of the illness dysphagia is found in up to 76% of patients [1-6], while dysphagic symptoms resolve in most of them within two weeks and persist in only a small number of subjects beyond six months [2-4]. Due to aspiration, malnutrition and dehydratation, dysphagia is associated with chest infection, prolonged hospital stay, institutionalisation and increased mortality [2,7-10].

Based on the results of the FOOD study early feeding via a nasogastric tube (NGT) is usually recommended as safe way of supplying nutrition in acute stroke patients [11]. There is, however, preliminary evidence that NGTs themselves interfere with swallowing physiology. Comparing NGT feeding with feeding via a percutaneous endoscopic gastrostomy (PEG) in a mixed collective of patients with neurological, ear, nose and throat or surgical problems Baeten and Hoefnagels reported swallowing difficulties in 17.4% of NGT-fed patients as opposed to none in the PEG group [12]. Furthermore, in a study of young and healthy volunteers Huggins and co-workers found different alterations of the swallowing mechanism with a NGT in place [13]. In contrast to this, two recent studies, the first dealing with post-acute stroke patients [14], the second examining a heterogeneous patient collective [15], did not observe a negative impact of the NGT on the act of swallowing.

According to a recent controlled trial early behavioral swallowing interventions are associated with a more favourable outcome in patients with dysphagic stroke [16]. In the light of this study it is principally desirable to start swallowing treatment with limited oral feeding during therapy even in stroke patients being temporarily fed via a NGT as early as possible.

The question of whether NGTs have an effect, if any, on dysphagia is hence of importance for acute stroke care. Due to the rapidly changing nature of dysphagia during the first two weeks after stroke [17], the above mentioned study of post-acute stroke patients is not easily extrapolated to the acute stage of the illness. In the present study we therefore investigated the impact of NGTs on swallowing function in acute stroke patients. In particular, two different topics were addressed. First, we examined how often pharyngeal misplacement of NGTs, in particular coiling of the tube in the pharynx, occurred and whether this led to worsening of dysphagia. Second, the impact of a correctly placed NGT on the swallow was explored.

## Methods

### Study design

This prospective study comprised of two parts called "Pharyngeal misplacement of the NGT: Frequency and consequences" and "Impact of a correctly placed NGT on the swallow". The first part was conducted as observational case series, the second used a pre-post design.

### Patients

One-hundred stroke patients were included in the first and 25 in the second part of the study. These consecutive patients were recruited between September 2006 and June 2007. All patients were admitted to our stroke unit within 24 hours of symptom onset. Exclusion criteria were severely decreased consciousness and unstable medical conditions such as severe pneumonia or decompensated congestive heart failure. Stroke severity was measured using the National Institute of Health Stroke Scale (NIH-SS) [18]. The study was approved by the local ethics committee and written informed consent was obtained from all subjects, or their next of kin, in case that the patient's communication was impaired.

### Clinical dysphagia screening

On admission to our stroke unit a dysphagia screening was performed in all patients [7]. In brief, the water swallowing test assessed the patient's ability to drink 5 ml (first step) and 50 ml (second step) of water[19]. Subjects who drank the water without cough or wet/hoarse voice were considered normal. Additionally the swallowing provocation test was used to evaluate the swallowing reflex [20]. The test requires the injection of 0.4 ml (first step) and, if necessary, 2.0 ml (second step) of distilled water into the pharynx through a small nasal catheter. As suggested by Teramoto and colleagues, this test was judged to be normal if the latency of swallowing after either of the water injections was less than three seconds [21,22]. Patients who failed at this clinical screening were considered to be at risk of aspiration and received a NGT.

### Fiberoptic endoscopic evaluation of swallowing (FEES)

The examination was carried out with an Olympus ENF-P4 laryngoscope attached to a camera and a color monitor. All examinations were videotaped. A neurologist experienced in using FEES and a speech-language pathologist (S.O.) jointly completed all FEES procedure. The standard FEES protocol was followed [23,24] with slight modifications as was described previously [25]. In brief, patients were evaluated at bedside on the local stroke unit in an upright position. The laryngoscope was passed through the most patent naris without administration of a topical anesthetic or vasoconstrictor to the nasal mucosa. The base of the tongue, pharynx and larynx were viewed. Before the presentation of any bolus, the patient's secretion level was noted and classified as "no pooling of secretions", "pooling without penetration/aspiration" and "pooling with penetration/aspiration". For evaluation of swallowing, the endoscope was placed in the high position above the epiglottis before and during the swallow to evaluate premature spillage and delayed swallowing reflex. After the swallow, the endoscope was advanced to the low position just above the vocal folds in order to evaluate penetration, defined as any material entering the laryngeal vestibule but remaining at or above the level of the vocal cords, or aspiration, defined as any material entering the airway below the vocal cords. If penetration or aspiration occurred the presence of protective reflexes was noted [26]. Following the procedure suggested in a previous study [23], the first food consistency introduced was puree, followed by liquid, and then white bread. All food was dyed with blue food coloring for contrast and was given in boluses of approximately 3 ml. Each food consistency was given three times and the worst result according to a simplified five-point penetration-aspiration scale (no penetration or aspiration, penetration with protective reflex, penetration without protective reflex, aspiration with protective reflex, aspiration without protective reflex) was noted. The endoscopist was free to terminate the examination at any time the patient's safety, seemed to be endangered, for example due to massive aspiration.

In the first part of the study patients with a NGT already in place were evaluated by means of FEES within 24 hours after tube placement. Apart from studying swallowing physiology, the examination focused on the position of the NGT within the pharynx. If a misplacement, like tube coiling, occurred this was corrected by cautiously pulling back the NGT with the endoscope left in place. Swallowing was reassessed thereafter.

In the second part of the study another subset of patients was studied twice within close succession, i.e. with and without a correctly placed NGT. Both examinations were usually carried out directly one after the other and were at most 1 hour apart. For pragmatic reasons, only patients were recruited in whom either the NGT could be removed during investigation because of a substantial improvement in swallowing function, or a NGT had to be placed due to newly recognized dysphagia. To reduce an expectation bias endoscopic examinations were videotaped and analysed off-line in random order by two independent raters (R.D., T.W.), so that they did not directly compare each single patient with and without a NGT.

### Nasogastric tubes (NGTs)

On our ward we use flexible silicon tubes (without stylet) with diameters of either 4.7 millimeters (14 charriere) or 5.3 millimeters (16 charriere).

### Statistical analysis

Statistical analysis was carried out with STATISTICA^® ^for WINDOWS^®^. In univariate analyses, the χ^2 ^test was used for categorical data and the t-test for continuous data.

## Results

In the total cohort of 125 patients there were 66 women and 59 men aged on average 70.0 ± 13.3 years. Their mean NIH-SS was 12.2 ± 5.3 points. 105 patients suffered an ischemic stroke and 20 patients presented with a hemorrhagic stroke. As is outlined in table 1, patients included in the first part of the study were slightly younger and there was a smaller proportion of women than in the second part, while the other epidemiological and clinical variables were equally distributed between both groups. FEES was carried out 3.6 ± 2.1 days after stroke.

**Table 1:** Epidemiological and clinical variables and stroke subtypes of the study collective.

	First Study N = 100	Second Study N = 25
Age (yr)	69.2 (13.1)	73.1 (13.9)
NIH-SS	12.1 (5.3)	12.3 (5.3)
Ischemic stroke (%)	84	84
- ACI	60	72
- PCI	20	4
- combined	4	8
Hemorrhagic stroke	16	16
- Hemispheric	11	16
- Brainstem	5	0

Where appropriate, means (standard deviations) are given. Yr = years, NIH-SS = National Institute of Health Stroke Scale, ACI = anterior circulation infarction, PCI = posterior circulation infarction

### First part of the study – pharyngeal misplacement of the NGT: Frequency and consequences

**Figure 1:**
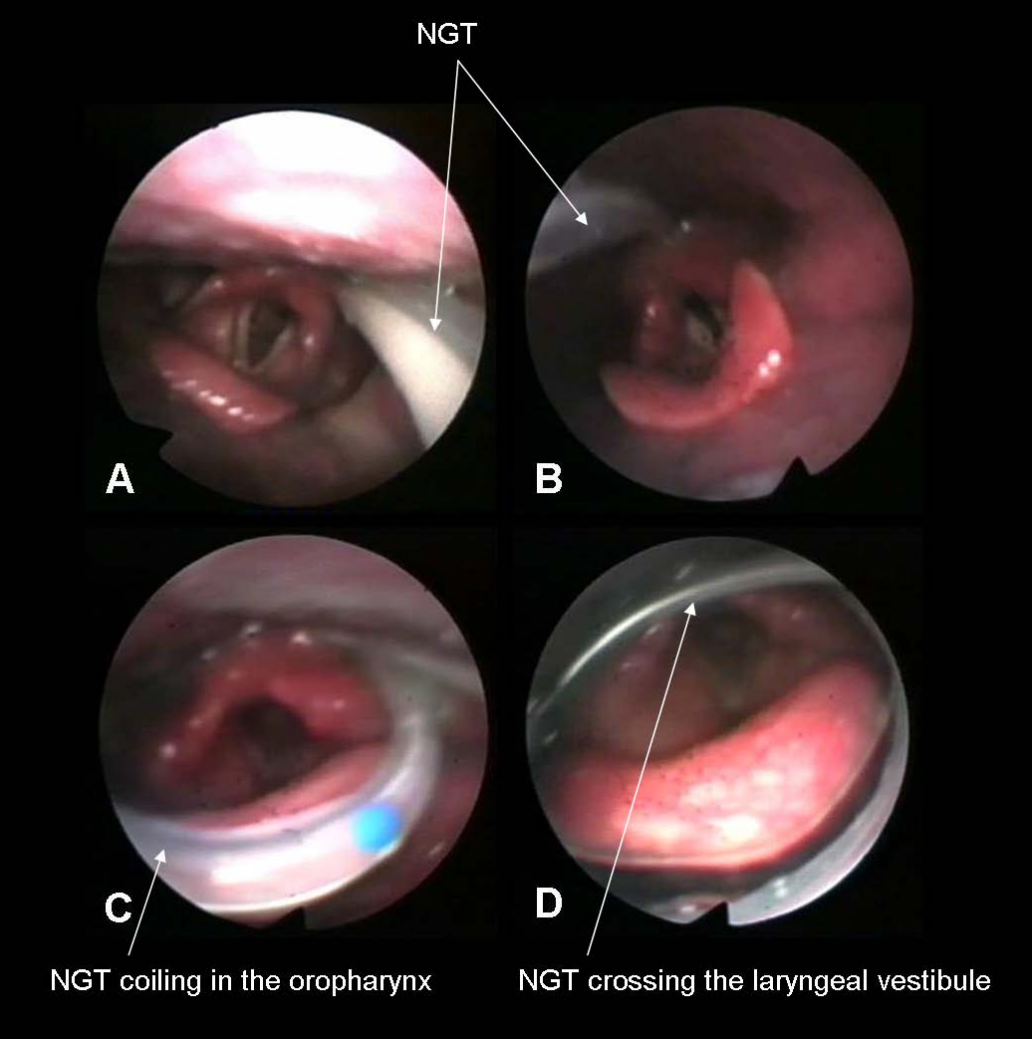
Different types of NGT position. A) Normal position along the lateral pharyngeal wall; B) Medial position with variable contact to the arytenoids; C) NGT coiling in the oropharynx; D) NGT coiling in the hypopharynx with crossing the laryngeal vestibule.

In 87 of the total of 100 patients the NGT took the appropriate course along the lateral pharyngeal wall down into the esophagus (Fig. 1A). In 8 patients the NGTs were placed more medially with variable dorsal contact to the arytenoids (Fig. 1B). In 5 patients misplacement of the NGT was noted consisting of its coiling within the pharynx. Tube coiling occurred at different pharyngeal levels and was found in the oropharynx (fig. 1C) as well as in the hypopharynx (fig. 1D). In all five cases the NGT looped around the epiglottis, in 3 patients the NGT also crossed the laryngeal vestibule thereby contacting the arytenoids (fig. 1D).

Signs of dysphagia did not change with correction of the 8 medially placed NGTs. Before and after correcting NGT placement, spillage of puree with penetration occurred in 5 patients. Three patients who swallowed puree without penetration or aspiration showed severe spillage resulting in penetration when being given fluids with either tube position. Postdeglutitive problems as possible consequences of upper esophageal sphincter dysfunction were not seen in any of these 8 patients. In contrast to this, pharyngeal coiling led to worsening of dysphagia in all five patients (table 2). With the misplaced tube being uncorrected, three patients, when being given puree, showed severe spillage leading to predeglutitive penetration in two and aspiration in one patient. After correcting the tube position spillage was markedly reduced in two – with one of them still showing penetration, and completely abolished in the third one. The other two patients showed only mild spillage when being exposed to puree but due to impaired pharyngeal contraction and incomplete epiglottis inversion intra- and postdeglutitive penetration occurred and pharyngeal residues were seen. After correcting the tube position mild spillage persists but intra- and postdeglutitive problems were not encountered any more.

**Table 2:** Changes of swallowing characteristics in 5 patients with pharyngeal misplacement of the NGT after correcting the tube position.

		Spillage	Residues	Penetration	Aspiration
Patient 1	Malpositioned NGT	++	-	+	-
	Corrected NGT	+	-	-	-
Patient 2	Malpositioned NGT	++	-	+	-
	Corrected NGT	+	-	+	-
Patient 3	Malpositioned NGT	++	-	-	+
	Corrected NGT	-	-	-	-
Patient 4	Malpositioned NGT	+	+	+	-
	Corrected NGT	+	-	-	-
Patient 5	Malpositioned NGT	+	+	+	-
	Corrected NGT	+	-	-	-

+ = moderate, ++ = massive

### Second part of the study – impact of a correctly placed NGT on the swallow

**Figure 2:**
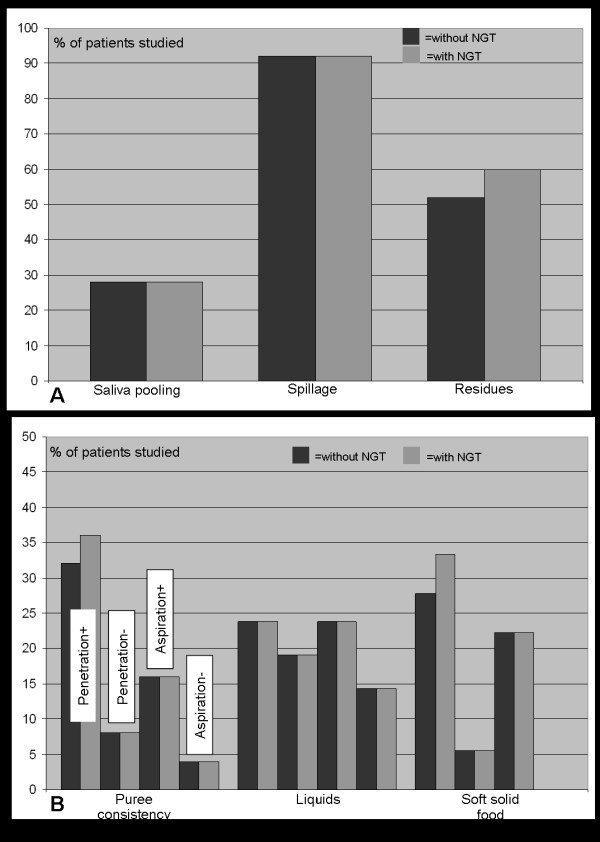
Main findings of FEES. Penetration+ = Penetration with protective reflex; Penetration- = Penetration without protective reflex; Aspiration+ = Aspiration with protective reflex; Aspiration- = Aspiration without protective reflex. The columns related to liquids and soft solid food are arranged in the same order as those related to puree.

In the second part of the study 18 of 25 patients were examined first without and then with a correctly placed NGT, in the other 7 patients the examination took the opposite course. During FEES all patients received puree, and 21 were given liquids, and 18 were exposed to soft solid food. As is summarized in figure 2A, salient endoscopic findings were not significantly altered by the presence of the NGT. Under both conditions saliva pooling without penetration or aspiration was observed in 28% of patients and spillage was found in 92% of them. In two patients who did not show residues without a NGT, those were observed with a NGT in place. However, since in both these cases swallowing material got stuck to the tube and remained in the sinus pyriformes after the swallow still attached to the NGT, these residues were not caused by a tube-related worsening of dysphagia but by a mechanical interference of the NGT with the swallowing material. The number of penetration- and aspiration-events across different food consistencies was also only insignificantly different between the two swallowing conditions. While swallowing of liquids was entirely unaltered by the presence of a NGT, one patient each with a safe swallow in the no-tube condition, showed penetration of puree and semisolid food respectively with a NGT in place (figure 2B). Again this result was due to the above mentioned mechanical interference of the NGT with the food bolus.

## Discussion

The most important finding of this study was that a correctly placed NGT did not alter salient findings of endoscopic swallowing examination in acute stroke patients. Except for two cases, in which swallowing material got stuck to the NGT, remained in the pyriforms and penetrated into the laryngeal vestibule after the swallow, no changes of the amount of penetration and aspiration were noted with the NGT in place as compared to the no-tube condition. This result expands on the study of Wang and colleagues [14], which examined 22 stroke patients about three weeks after disease-onset by means of videofluoroscopy. In that investigation the placement of a NGT did not affect temporal and non-temporal measures of swallowing. Our results are also in keeping with a recently published large and methodologically different study [15]. Leder and Suiter carried out FEES in 1260 inpatients with a variety of different diseases, among them 214 patients with acute stroke. Comparing groups of patients with and without a NGT in place they found no differences in aspiration status between them.

As second main finding, pharyngeal misplacement of the NGT was identified in 5 of 100 patients. In all these patients the NGT looped around the epiglottis and in three of them the NGT also crossed the laryngeal vestibule thereby contacting the arytenoids. All patients showed worsening of dysphagia caused by the misplaced NGT with an interindividually variable increase of pre-, intra-, and postdeglutitive penetration. Interestingly, a medial course of the NGT with variable contact to the arytenoids observed in 8% of patients did not alter swallowing physiology.

Previous studies examining the frequency of malpositioned NGTs mainly focus on inadvertent placement into the respiratory tract. In a review of more than 2000 tube insertions, Sorokin and Gottlieb identified 50 documented cases of NGTs entering the bronchial system corresponding to a incidence rate of below 2,5% [27]. Other complications are reported less frequently and are mainly the subject of case reports or small case series. Thus, if a NGT is not moved forward far enough it may end in the distal esophageus. To start tube feeding via such a malpositioned NGT may increase the risk of regurgitation and consecutive aspiration[28]. Inadvertent placement of a NGT into the brain of patients with traumatic defect in the cribriform plate fortunately happens very rarely, although reports of this complication still occur [29]. Taken together, pharyngeal coiling is probably the most frequent type of NGT misplacement. Since this anatomical region is not assessable by conventional chest radiography usually done to verify NGT position, it has most likely been underreported so far.

When interpreting the findings of the present study the following methodological limitations need to be addressed. First, although in both studies endoscopic examinations were videotaped and analysed off-line in random order an expectation bias could not be fully ruled out since in most cases the presence or absence of a NGT can be deduced from the video. Furthermore, the second part of the study did not use a randomized order of investigating the tube vs. no-tube condition, which might have introduced an order effect.

## Conclusion

From the clinical point of view, the following consequences may be drawn from the present study. First, since correctly placed NGTs did not cause worsening of dysphagia they are no principle obstacle to start oral feeding in affected patients. Therefore, dysphagic stroke patients without endoscopically proven overt risk of aspiration may receive limited amount of oral food, for example as part of early swallowing therapy, even with a NGT still in place [25]. Second, since pharyngeal misplacement was only found in 5% of patients, FEES may not necessarily be performed to rule out this condition in all tube fed stroke patients prior to the start of oral intake. However, because pharyngeal coiling – even if rare – may cause a worsening of dysphagia and predispose patients to penetration or aspiration with possible devastating consequences, a close clinical monitoring looking for disturbed swallowing and aspiration should initially be performed in these situations.

## Abbreviations

NGT: Nasogastric tube; PEG: percutaneous endoscopic gastrostomy; NIH-SS: National Institute of Health Stroke Scale; FEES: Fiberoptic endoscopic evaluation of swallowing.

## Competing interests

The authors declare that they have no competing interests.

## Authors' contributions

RD, TW, CH, SO, CK and IK were involved in the FEES. RD, TW, WRS and EBR designed the study protocol. RD, TW and MR performed statistical analysis. RD and TW wrote the manuscript. MR, WRS and EBR read previous drafts of the published manuscript and made substantial improvements. All authors read and approved the final manuscript.

## Pre-publication history

The pre-publication history for this paper can be accessed here:


